# Towards understanding and synthesis of contact-rich anthropomorphic motions through interactive cyber-physical human

**DOI:** 10.3389/frobt.2022.1019523

**Published:** 2022-12-01

**Authors:** Eiichi Yoshida

**Affiliations:** Department of Applied Electronics, Faculty of Advanced Engineering, Tokyo University of Science, Tokyo, Japan

**Keywords:** humanoid robotics, cyber-physical human, contacts, motion understanding, whole-body motion synthesis, optimization

## Abstract

This article presents perspective on the research challenge of understanding and synthesizing anthropomorphic whole-body contact motions through a platform called “interactive cyber-physical human (iCPH)” for data collection and augmentation. The iCPH platform combines humanoid robots as “physical twins” of human and “digital twins” that simulates humans and robots in cyber-space. Several critical research topics are introduced to address this challenge by leveraging the advanced model-based analysis together with data-driven learning to exploit collected data from the integrated platform of iCPH. Definition of general description is identified as the first topic as a common basis of contact motions compatible to both humans and humanoids. Then, we set continual learning of a feasible contact motion network as the second challenge by benefiting from model-based approach and machine learning bridged by the efficient analytical gradient computation developed by the author and his collaborators. The final target is to establish a high-level symbolic system allowing automatic understanding and generation of contact motions in unexperienced environments. The proposed approaches are still under investigation, and the author expects that this article triggers discussions and further collaborations from different research communities, including robotics, artificial intelligence, neuroscience, and biomechanics.

## 1 Introduction: iCPH—Interactive cyber-physical human platform

Humanoid robots are expected to help humans in various scenarios owing to their versatility and anthropomorphic shape making it easy to adapt to environments designed for humans. While they still need improvements in reliability and safety, we believe that they are steadily making progress to be integrated into our society in the future. On the other hand, it can have another scientific role as a “physical twin” of human in research areas that include modeling and understanding human motions, more widely behaviors. One example is to evaluate wearable devices in place of human subjects (Yoshida et al., 2018) by a humanoid robot reproducing measured human motions. Indeed, human motions involving more complex contacts including surface contacts have still a lot to be investigated: what humans optimize while they are moving, how human motions can be predicted, and what can be the optimal robot motions when interacting with humans.

In this aspect, human motion analysis has recently made remarkable progress based on model-based technologies such as motion optimization, musculo-skeletal (MS) analysis, and dynamic simulation. Nevertheless, interactions with objects and environments through complex contacts need further investigation. We actually still have a long way to achieve automatic understanding and generation of anthropomorphic whole-body (WB) motion sequences involving multiple contacts.

**FIGURE 1:**
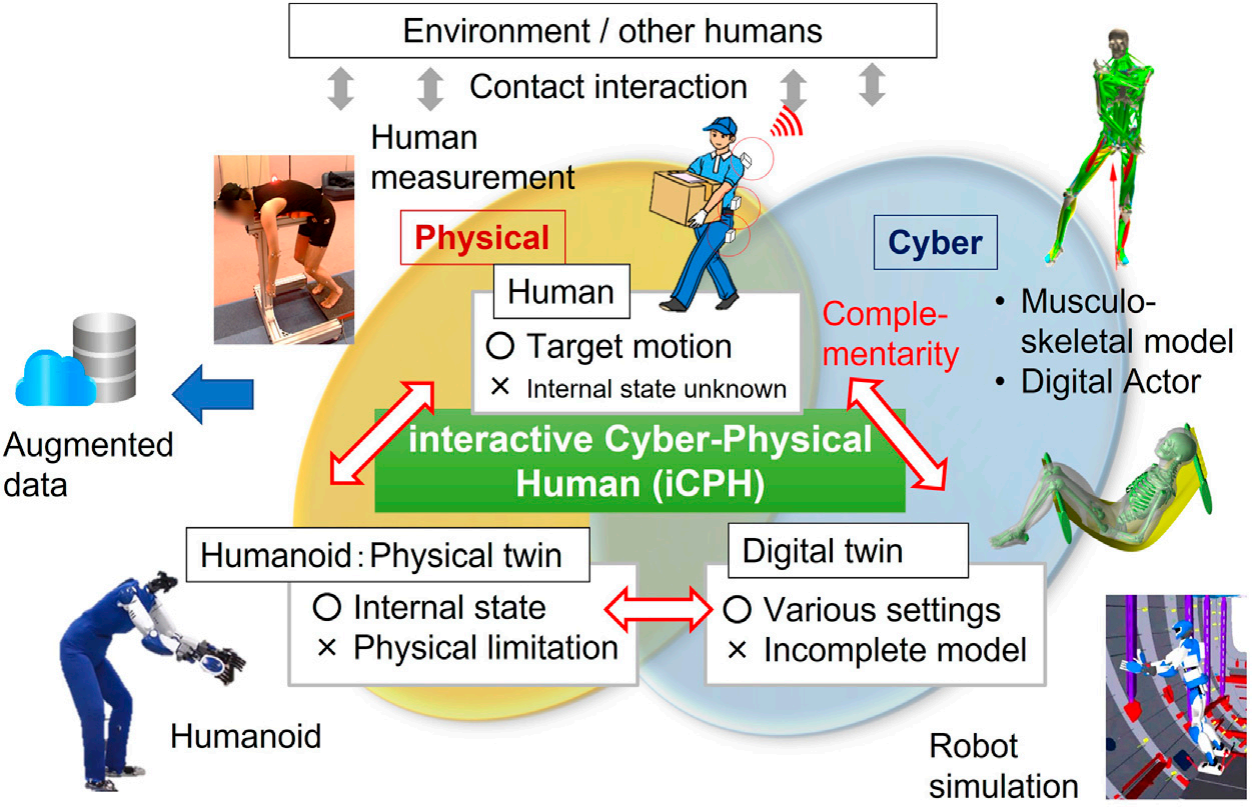
Platform of interactive cyber-physical human (iCPH) for investigation of contact-rich whole-body motion of anthropomorphic systems.

Humanoid robots as physical twins are exploited for real-world validation, while digital twins can serve for the improvement of analysis and simulation quality, benefiting from advanced machine learning techniques that can deal with increasing available datasets of human motions. Humanoid robots and digital actors therefore can be leveraged together in a complementary manner as “interactive cyber-physical human (iCPH)” for this purpose, since it is still difficult to measure the internal control signal of humans, whereas such biomechanical approaches like musculo-skeletal systems have been developed (Figure 1). Placing human measurement and model-based robotic approaches as an important basis, our challenge is to overcome this difficulty also by exploiting a powerful machine learning framework to benefit from a large dataset of human motions.

Consequently, we can take advantage of humanoids’ ability to interact with the physical world to refine and validate the model of the motion strategy and controller, as well as digital actors’ flexibility to change many parameters to simulate and learn motions with various shapes, dimensions, and physical models in different environments. Then, we hope to come up with a system that predicts and synthesizes human motions, notably motions involving complex contacts, in a variety of environments. As the cyber-physical human evolves, we expect it can be utilized to design ergonomic products, create robots that can support human comfortably by estimating human intention, and devise a humanoid robot that can coexist with humans naturally and safely in their proximity.

Table 1 summarizes the state of the art related to contact-rich whole-body motions in different anthropomorphic systems. Concerning human motions, we can see that datasets with measured whole-body contact forces, in addition to measurements of ground reaction force, are missing. The human motion network rarely deals with motions involving contacts. While digital human models are advanced in terms of surface contacts, their main purpose is to analyze the measured human motions, and motion synthesis needs to be further investigated through an understanding of human motion principles. Whole-body contacts have been intensively addressed in humanoid motion planning and control in view of the increasing variation of humanoids’ tasks. However, whole-body motion with multiple surface contacts has hardly been studied for humanoid robots, limiting the variety of their motions.

**TABLE 1:** Related works on contact-rich whole-body motions by cyber-physical humans. The mark “NA” means the item is not applicable to the category.

Category		Reference	WB motion		WB contacts: detect (*D*) and force (*F*)	
			Analysis/input	Synthesis	Point	Surface
Human motion	Measured data	CMU	*✓*MoCap	NA	*✓D* Estimated	*✓ D* Estimated
		AMASS	*✓*Integrated	NA		
		BEHAVE	*✓*RBGD camera	NA		
		RICH	*✓*RGBD camera	NA		
		HuMoD	*✓*MoCap	NA	*✓F* Foot force plates	
		MMM	*✓*MoCap	NA	*✓F* Measured some	
	Network	Motion Graph	*✓*	*✓*Replay	*✓D* Classified	
		Motion Net		*✓*Feedforward		
		Loco-manip		*✓*Classified		
		Taxonomy				
		HO-GCN	*✓*MoCap		*✓F* Estimated	
		Crystal Ball		*✓*Symbol learned		
Digital human	3D mesh	Dhaiba	*✓*	*✓*Inverse Dyn	*✓F* Estimated	*✓F* Estimated
		THUMS	*✓*			
		Mass-Spring	*✓*			*✓F* Estimated
	MS model	SIMM	*✓*		*✓F* Estimated	
		AnyBody	*✓*		*✓F* Estimated	
		Realtime MS	*✓*		*✓F* Estimated	
Humanoid motion	Planning/ trajectory	HRP-4	NA	*✓*Retargeting	*✓F* Retargeting	
		HRP-2	NA	*✓*Best-first	*✓F* Predefined	
		HRP-5P	NA	*✓*Feasible region	*✓F* Predefined	
		TORO	NA	*✓*Constrained	*✓F* Predefined	
		Two-stage	NA	*✓*Constrained	*✓F* Automatic simplified	

	Control	HRP-2	NA	*✓*Min. torque	*✓F* Minimization	*✓F* Interactive upper-body
		HRP-5P	NA	*✓*QP static	*✓F* Minimization	
		TORO	NA	*✓*QP dynamic	*✓F* Minimization	
		Soft Robot (Honda)	NA	*✓*Retargeting		
	
		REEM-C (TUM)	NA	*✓*Torque resolving		*✓F* Interactive upper-body
		

As can be seen, motions of anthropomorphic systems with complex contacts, especially surface contacts, need deeper research combining all the aspects in the table. It is important to unify complementarily the insights from data collection with contact-rich human motions, modeling of a strategy for human motion synthesis with a digital twin, and planning and control methods for humanoid whole-body contact motions. This is the motivation of the proposed cross-platform research using a cyber-physical human framework in Figure 1, and as there are still a lot to carry out in this research perspective, interdisciplinary synergy from different communities is highly expected. This table will be revisited later in the next section.

One critical question that arises when creating such a cyber-physical human framework is how to deal with the physical difference between humans and humanoids. While they are indeed very different in many aspects like physical properties, link and actuator mechanisms, and contacts, they have a common anthropomorphic structure, which we identified as the starting point. The general descriptor presented later is meant as one of the elements connecting humanoids and humans. The main reason why humanoids have difficulty in generating smooth multi-contact motions like humans is that humanoids are not as flexible and agile as humans. However, we believe that the humanoids can learn the general motion strategy for whole-body planning and control from humans. We are furthermore motivated to provide humanoids with humans’ intelligence to naturally select contact points in an unexperienced situation and move by appropriately managing the contact forces and balance.

We also need to be aware of differences in the physical property of contacts. Point contacts are mainly considered in most of the cases for humanoids, whereas humans take advantage of surface and soft contacts. Therefore, we plan to measure surface contact forces for humans with distributed tactile skin sensors (Cheng et al., 2019), which can also be equipped on the surface of humanoid robots with a flexible covering material. Since neither human data acquisition nor human whole-body motion with surface contacts has been addressed, we would like to fill the gap using this experimental study. Once this basic underlying human motion mechanism is investigated, we expect that it can be adapted to humanoids by taking into account the physical differences. Even though the findings from human motion analysis unfortunately revealed minor contributions to humanoid motions as a result, it will be a step forward to understand the human motion strategy, which is useful for behavior prediction.

This cyber-physical human framework can be explained by an analogy with the cyber-physical system for automatic driving. Experimental data from human drivers are important to model the driving behavior, but that cannot cover all the cases. Then, simulations in cyber-space with different parameters or situations help the design of automatic driving controller to complement the missing domain of experiments in physical space. The accuracy of human behavior model can be improved by comparing the resultant behavior of human and simulation, in the same way as humanoid robots contribute to improvements of the simulation model. Once the simulated driver becomes accurate enough, a lot of control methods can be tested only in cyber-space. In the case of cyber-physical human, one of the challenges is to establish the general motion strategy that allows synthesizing whole-body contact motions in unexperienced environments. Similarly, an automatic driving controller may have to cope with many unexpected situations while driving among full of pedestrians or close to an ambulance. The necessity of learning to obtain such a higher-level strategy in the cyber-physical system can be understood in this analogical discussion.

This article is to introduce challenges and stimulate discussion leading to collaborations rather than reporting obtained results. After overviewing the challenges to be tackled using the iCPH platform in Section 2, they are addressed in detail as scientific problems related to contact-rich motion understanding and synthesis in Section 3: Generic descriptor of contact motion (3.1), Continual learning of the contact motion network (3.2), and Optimization, prediction, and synthesis through symbolization (3.3). Possible applications and summary are provided in Section 4.

## 2 Challenges for contact-rich motions through iCPH

The basic question comes from lack of a generic methodology for the analysis or prediction of anthropomorphic motion involving complex contacts. Since an anthropomorphic system is not fixed, it moves itself and other objects through contacts with its environments. In our daily life, we naturally do such motions as “setting on a chair by placing hands on the table,” “pushing a cart carrying heavy object,” or “going through a narrow space by supporting contacts between body parts and handrail or walls.” Recently, humanoid robots have achieved multi-contact motions in complex environments with robots HRP-2 (Lengagne et al., 2013), TORO (Henze et al., 2016), and HRP-5P (Kumagai et al., 2021). While they propose sophisticated planning methods and control strategies, multiple surface contacts are out of their scope. Recently, humanoid robots covered with tactile skins to recognize surface contacts (Cheng et al., 2019; Kaplish and Yamane, 2019) have been developed and demonstrated their high interactivity with humans, with possible future developments towards whole-body (see Table 1).

Human motion analysis has also made progress, leading to musculo-skeletal analysis allowing to estimate activities of hundreds of muscles (Nakamura et al., 2005; Ayusawa and Nakamura, 2012), as well as some commercial products such as SIMM and AnyBody (Nunes et al., 2015). Simulation tools with digital human models like Dhaiba (Endo et al., 2014) and THUMS (Iwamoto et al., 2002) integrate contact models to simulate products with the dynamic reaction of the human body. They can simulate passive behaviors of humans in case of surface contacts (Yoshiyasu et al., 2015) using a mass-spring model. However, the contact sequence is planned or annotated usually manually based on experiences. On the other hand, especially in the computer vision and graphics field, many datasets incorporating contacts are available, for example, BEHAVE (Bhatnagar et al., 2022), RICH (Huang et al., 2022), HuMoD (Wojtusch and von Stryk, 2015), and MMM (Mandery et al., 2016). While they are very useful to broaden the range of motion creation for animation or digital human, data with contact forces need to be reinforced, and their physical plausibility is to be improved for robotic motion generation yet.

The motion with a known contact sequence can be predicted using a model-based approach. In contrast, we need to solve an inverse problem for a humanoid robot or digital actor in order to generate whole-body motions involving multiple contacts to achieve a given task in a complex environment, which is very difficult to solve. The main difficulty comes from the fact that discrete changes in contact constraints are applied to dynamic continuous motions, making the possibility of combined motions increase exponentially. In order to generate motions that do not fail, we should carefully design the contact sequence and force distribution from these practically infinite possibilities, by considering the feasibility by a whole-body dynamic controller. Due to this high complexity, this problem remains open even with the advanced machine learning technology.

We believe an important key to this challenge of the inverse problem is a data-driven approach that makes use of human motions that are known to be feasible. Consequently, the motion capacity of robots to cope with complex environments can be drastically extended to execute various tasks. Nevertheless, the data on anthropomorphic contact-rich motions are still unavailable as the methodology for collection of such data has not been well established yet. We therefore need to build up this integrated iCPH platform for data acquisition, together with a new comprehensive data-driven theoretical framework for understanding and synthesizing anthropomorphic contact-rich motions, to go beyond the conventional methods by unifying both model-based and machine learning methodologies.

**FIGURE 2:**
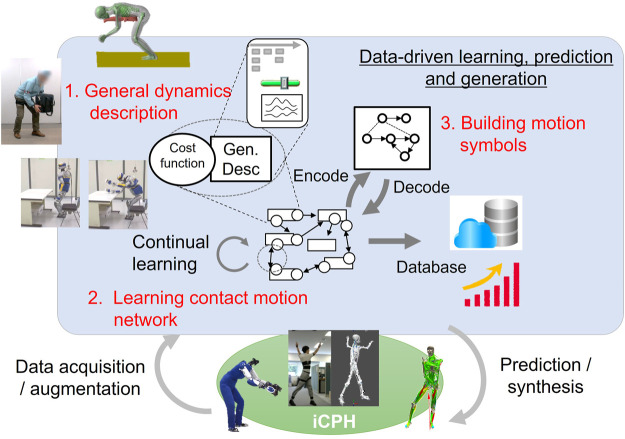
Three main challenges addressed in this article based on the iCPH platform: (1) general dynamics description of contact motions, (2) learning the contact motion network, and (3) building motion symbols.

We have identified mainly three challenges, as illustrated in Figure 2. The first important element we recognized for this unified theoretical framework is a common generic description of contact motions for anthropomorphic systems. We will first discuss how this descriptor should be devised so that it can express contact motions both in a compact and generic manner with minimum parameters and constraints. Combining this descriptor with a dynamic equation, different contact motions can be written in a standardized way.

On this basis, we tackle the second challenge of learning a network of feasible contact motions. Instead of trying to learn the behavior of the entire system, including the dynamic model, the control strategy of measured human motions is abstracted by applying inverse optimal control (Mombaur et al., 2010; Carreno-Medrano et al., 2019; Ishida et al., 2021) and analytical gradient computation (Ayusawa and Yoshida, 2018). The resultant estimated cost function is used in combination with the descriptor to characterize contact motions. This pair of cost function and descriptor corresponds to a feasible transition from one contact state to another as shown in Figure 2. Recently, research studies have been very actively made on learning robotic manipulation by deep learning (Gu et al., 2017; Yang et al., 2017). Its application to whole-body contact motions by anthropomorphic systems, however, is still a difficult problem due to its high dimensionality and complexity. In contrast, in our proposed framework, the merit of machine learning can be effectively exploited to learn the network of feasible contact motions since the descriptor already has a compact form with a reduced dimension. In the computer graphics area, usage of the large-scale human motion database mentioned previously has been addressed for the synthesis of natural human-like motions as a motion network (Holden et al., 2016), Motion Graphs (Kovar et al., 2008), and for classification of contact motions toward taxonomy of loco-manipulation (Borras and Asfour, 2015) and prediction of object manipulation by HO-GCN (Wan et al., 2022). They provide an advanced insight into natural human motions but concerning whole-body motions of humans or humanoid robots involving complex contacts, they are out of scope of those studies and the related datasets themselves are not widely available yet.

Finally, toward prediction and automatic synthesis of contact motions, we aim at higher abstraction by symbolizing them through vector quantization (VQ) learning. Symbolic understanding has been addressed based on probabilistic methods like the hidden Markov model (HMM) in their Crystal Ball system (Takano and Nakamura, 2015), but the high-level expression of contact motions is still challenging due to its complexity. At the end of this article, some possible applications that can arise from the outcome of this research are also discussed.

## 3 Data-driven whole-body contact motion synthesis

**FIGURE 3:**
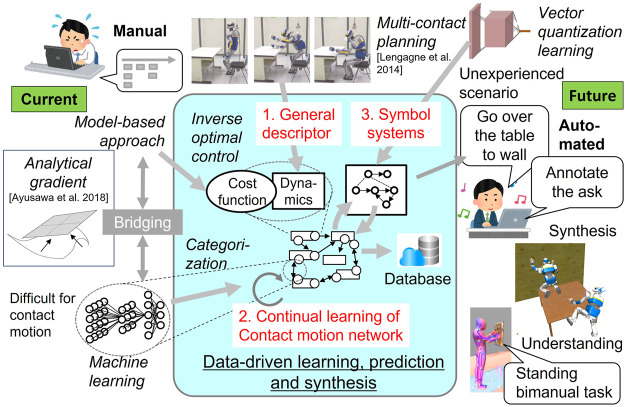
Current technologies (noted in *italics*) to tackle the challenges and expected outcomes of this research. The analytical gradient bridges the model-based approach and machine learning to obtain the contact motion network. Symbolic representation is obtained based on vector quantization (VQ) learning for high-level automatic understanding and synthesis of contact motions instead of manual annotation and planning.

Figure 3 depicts how the proposed framework is related to existing research including ours and also expected outcomes. Based on a general description of contact motions, a model-based approach such as inverse optimal control is used to estimate cost function. As presented later, analytical gradient computation (Ayusawa and Yoshida, 2018) serves as a bridging block unifying model-based and learning methods in a complementary manner to enable continual learning of contact motions without embedding the model itself in learning. VQ learning further pushes the abstract understanding forward to create high-level symbols toward automatic annotation or synthesis of contact motions in unexperienced situations.

### 3.1 Generic descriptor of contact motion

**FIGURE 4:**
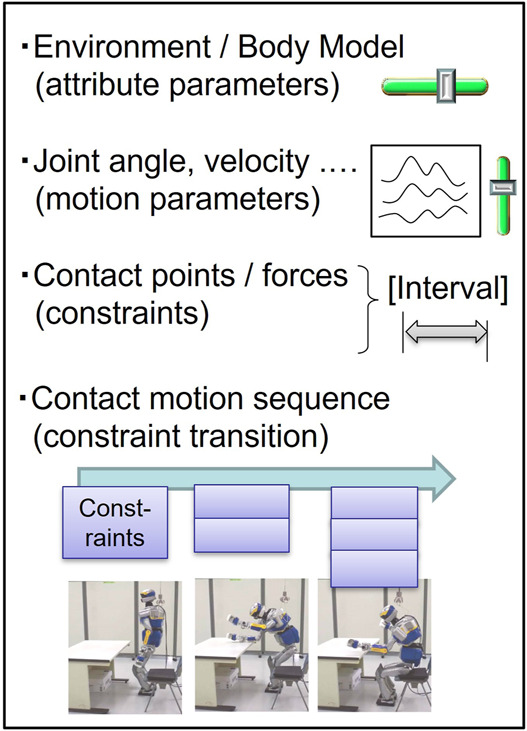
Generic descriptor of anthropomorphic contact motions, including body and environment models, motion parameters, contact constraints, and their sequences. This compact expression allows the standard expression for the upcoming learning.

The main objective of defining the descriptor is to represent discrete changes of contact constraints for continuous motions in a generic manner, without excessive simplification losing essential properties (Figure 4).

We are still investigating this general description, but we have derived the following specifications based on our experience. The descriptor shall first include attribute parameters that express the shape and structure with respect to the body coordinate (Yoshiyasu et al., 2014; Yoshiyasu et al., 2019) accompanied with various contacts on points or surfaces. Then, a contact sequence can be formulated together with the temporal transition, including additions and removals of inequality constraints, corresponding to force distribution to multiple contacts. The resultant motion of the contact-rich anthropomorphic system can be described in a compact standard form as a combination of a dynamic differential equation and the contact transition.

### 3.2 Continual learning of the contact motion network

**FIGURE 5:**
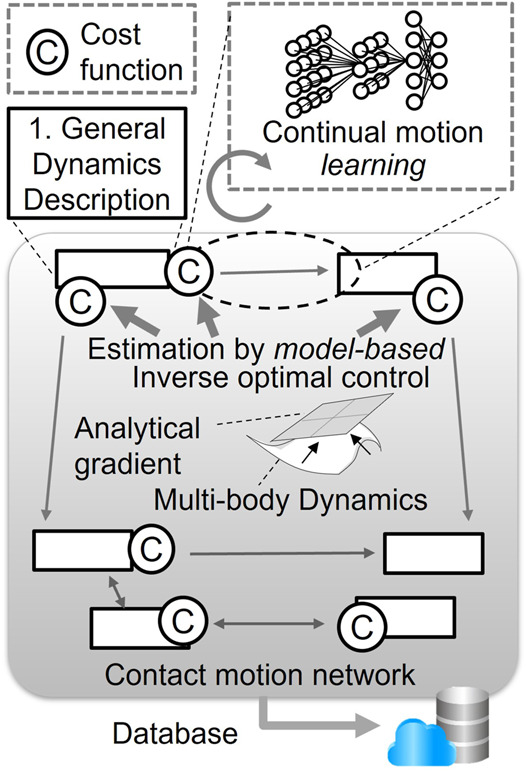
Continual learning of the contact motion network unifying model-based and machine learning approaches. Cost function of contact motion is first identified based on efficient inverse optimization benefiting from analytical gradient computation. By the abstract expression of contact motion with the combination of generic dynamics description and cost function, interrelation of the contact sequence can be learned without embedding known dynamic models.

The schematic view of contact network learning is illustrated in Figure 5. Dimension reduction of the problem can be achieved by describing the contact motions by the dynamic differential equation where the joint trajectory is expressed with a small number of parameters like B-spline, together with the transition of contacts described by representative points and forces with certain approximation precision. When optimal control is applied with a given cost function, the anthropomorphic system generates a motion to transit to the desired status of its body and contacts. During this process, the gradient of the cost function and constraints should be computed. The aforementioned analytical gradient computation (Ayusawa and Yoshida, 2018) allows efficiently computing the derivative of a cost function composed of various physical quantities with respect to the variance of the trajectory in motion optimization, which was difficult with conventional numerical differentiation. The generic description introduced previously helps to formulate the dynamic motion in a standardized form.

We are interested in identifying the cost function of human motions and then utilizing it for humanoid motion synthesis. Inverse optimal control (Mombaur et al., 2010; Ishida et al., 2021) has been applied to estimate the cost function under the hypothesis of humans make their motions by optimizing some criteria. The usual approach is to derive the weights of some known physical quantities such as energy, sum of joint torques, change rate of motion, velocity, or acceleration (Carreno-Medrano et al., 2019), as well as additional criteria related to contact forces. By applying inverse optimal control integrated with this analytical gradient to the measured contact motions, the basis of cost functions and their weights in optimization is estimated. These are considered to be highly abstract feature values characterizing the motion.

To this end, the cost functions have been extracted that generate motions from one contact status to another. We next intend to know how those motions are interrelated. For example, when moving in a narrow space using hands and back to support the body, what kind of cost function is needed to move the body smoothly to the next contact without losing balance? A machine learning scheme is considered to be a suitable solution as it learns categories based on differential relationships and has high affinity with the analytical gradient. It is expected to learn feasible contact motions efficiently without embedding with the known dynamic equation in the learning system itself. In this way, the network of a pair of constraints and cost functions generating contact motions without failure can be extracted from huge data through unsupervised learning. Moreover, with continual learning, such a network is expected to evolve in a sustainable way to cover wider contact motions with increasing data.

The resultant network represents how to transit from one contact state to another successfully and can be utilized as a database that is not just a set of independent motion data but also includes interrelation between them. It can be further enriched as a variable database according to different body parameters through data augmentation using motion retargeting (Di Fava et al., 2016; Ayusawa and Yoshida, 2017) between different bodies and also dynamic simulations under different conditions. This network will be exploited for symbolization presented in the following section.

### 3.3 Optimization, prediction, and synthesis through symbolization

This stage applies modified “vector quantization (VQ)” learning to the obtained network to get symbols as higher-level feature values. The expected benefit here is composing a kind of a language system of interrelated various contact motions to be able to generate optimal motions in unexperienced environments. Among several methods, VQ-VAE (Vector-Quantized Variational AutoEncoder) (van den Oord et al., 2017; Razavi et al., 2019) is one of the methods that possess high abstraction ability. The network learned previously expresses the transition of contact motions as the temporal sequence of the pair of constrained dynamics and estimated cost functions. Application of model-predictive control provides guidelines of a control strategy to reach the target state by utilizing the relationship between them in this network. The VQ-VAE is adapted in such a way that it can automatically identify discrete labels that consist of a variety of contact motions corresponding to those abstract control strategies. In our preliminary experiments (Sakai et al., 2021), we applied the VQ-VAE framework to Japanese “kendo” practice motion with a bamboo sword. As a result, this combined movement can be encoded with only two states to reproduce closely learned motion by decoding. Then, we can attribute to those states some symbols like “swing practice.”

We can benefit from our recent promising results of classification and labeling of human motions. Those discrete labels can be organized as “symbol” forming a language system that allows a high-level representation of various contact motions. In previous research (Takano et al., 2015; Takano and Nakamura, 2015), the HMM-based model learned motions such as “walking,” “nodding,” or “jumping,” but adding contacts made the problem intractable due to complexity as this method is already computationally intensive. By applying the vector quantization method to a subset of sequences in the learned network, also by taking advantage of analytical computation in 3.2, we expect to encode it with a small number of states to describe the motion sequence, like “sit on or stand up from a chair,” “pull a drawer,” or “climb a ladder.” The related study of taxonomy (Borras and Asfour, 2015) can also be utilized to organize those symbols.

**FIGURE 6:**
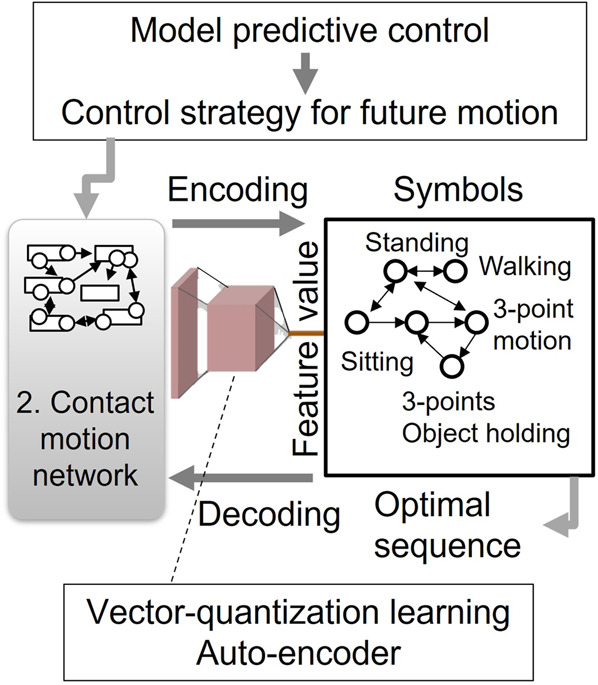
Symbolization of contact motions through VQ learning. Control strategies that are obtained from the network by model predictive control are encoded into high-level symbols. The optimal contact sequence for a given goal state is automatically synthesized using the symbols and decoded back to a whole-body contact motion.

Thanks to its flexibility of combining elementary labels, this symbol system has a potential to synthesize a wide variety of optimal contact motions ranging from simple walking to complex ones such as multi-contact motions in confined spaces, pushing a cart with heavy objects or whole-body object holding and dual-arm tool usage as shown in Figure 6.

This symbolic system facilitates planning of contact motions by using high-level commands by decoding symbols into optimal anthropomorphic whole-body motions in given environments, independent of body property parameters that are embedded in the general descriptor. The contact motion network and symbolized system together enable prediction of future motion outcomes in the range of several hundreds of milliseconds up to seconds, so as to deal with unexperienced environments. We expect to apply this basic technique to contact motion planning and online adaptation, as well as automatic annotation of complex contact motions.

## 4 Possible applications and article summary

**FIGURE 7:**
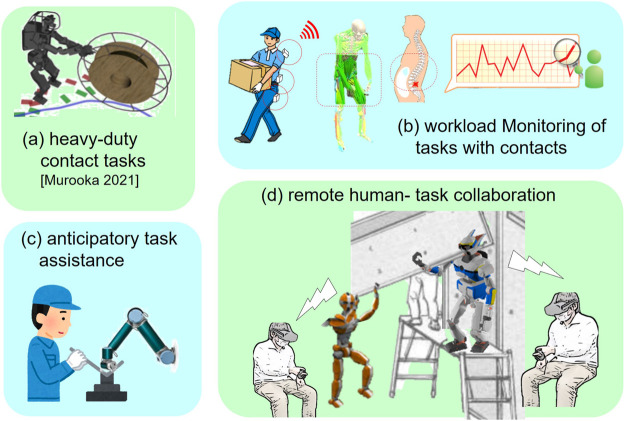
Possible applications of the proposed data-driven contact motion synthesis for humans and humanoid robots.

The established framework of data-driven synthesis of whole-body anthropomorphic contact motions is planned to be applied to various application areas, especially to automate manual tasks dependent on experienced experts, such as planning of contact transition and force distribution or contact annotation of measured human motions. Feedback from real-world tasks allows finding unaddressed conditions and issues to fill the gap between physical and cyber-space and thus make the proposed framework even more robust. The possible applications include the following, as shown in Figure 7.

(a) Online planning whole-body motion of a humanoid robot allowing to manipulate a heavy object (Murooka et al., 2021) or to execute tasks requiring uncomfortable posture in confined spaces, often can be seen in large-scale assembly in construction of airplanes (Kheddar et al., 2019), ships, or buildings.
(b) A monitoring system visualizing and anticipating physical workload of workers to prevent work-related diseases through musculo-skeletal analysis based on sensor information collected from embedded devices in clothes, for heavy-duty tasks in factories or in other industries.
(c) Assistive robots in a factory or caregiving scenario that can offer anticipatory physical support to operators and workers in charge of assembly tasks or patient transfer, by symbolically recognizing and predicting those whole-body contact-rich motions.
(d) Human–robot collaborative task execution such as large equipment installation or object transporting with an avatar robot whose contact sensing is shared with remote operators in telepresence virtual space.

This article on the research perspective discussed the challenges of data-driven understanding and synthesis of whole-body anthropomorphic motions involving frequent contacts. After introducing a general descriptor of contact motions, model-based analysis and machine learning are jointly utilized for continual learning of a network of feasible contact motions, thanks to the analytical gradient computation method. A symbolic language-like system is then derived by vector quantization learning that is capable of automatic synthesis and understanding of optimal contact motions. The challenges are still under investigation in the project the author is leading, expecting to trigger interdisciplinary collaboration and report the upcoming results in future publications.
